# Comparison of methods generating antibody-epitope conjugates for targeting cancer with virus-specific T cells

**DOI:** 10.3389/fimmu.2023.1183914

**Published:** 2023-05-16

**Authors:** Willemijn van der Wulp, Anna M. Gram, Boris Bleijlevens, Renate S. Hagedoorn, Can Araman, Robbert Q. Kim, Jan Wouter Drijfhout, Paul W. H. I. Parren, Richard G. Hibbert, Rob C. Hoeben, Sander I. van Kasteren, Janine Schuurman, Maaike E. Ressing, Mirjam H. M. Heemskerk

**Affiliations:** ^1^ Department of Cell and Chemical Biology, Leiden University Medical Center, Leiden, Netherlands; ^2^ Genmab, Utrecht, Netherlands; ^3^ Department of Hematology, Leiden University Medical Center, Leiden, Netherlands; ^4^ Division of Bio-organic Synthesis, Leiden Institute of Chemistry, Leiden University, Leiden, Netherlands; ^5^ Department of Immunology, Leiden University Medical Center, Leiden, Netherlands

**Keywords:** antibody-epitope conjugates (AECs), redirecting virus-specific T-cells, immunotherapy, targeted therapy, conjugation strategies

## Abstract

Therapeutic antibody-epitope conjugates (AECs) are promising new modalities to deliver immunogenic epitopes and redirect virus-specific T-cell activity to cancer cells. Nevertheless, many aspects of these antibody conjugates require optimization to increase their efficacy. Here we evaluated different strategies to conjugate an EBV epitope (YVL/A2) preceded by a protease cleavage site to the antibodies cetuximab and trastuzumab. Three approaches were taken: chemical conjugation (i.e. a thiol-maleimide reaction) to reduced cysteine side chains, heavy chain C-terminal enzymatic conjugation using sortase A, and genetic fusions, to the heavy chain (HC) C-terminus. All three conjugates were capable of T-cell activation and target-cell killing *via* proteolytic release of the EBV epitope and expression of the antibody target was a requirement for T-cell activation. Moreover, AECs generated with a second immunogenic epitope derived from CMV (NLV/A2) were able to deliver and redirect CMV specific T-cells, in which the amino sequence of the attached peptide appeared to influence the efficiency of epitope delivery. Therefore, screening of multiple protease cleavage sites and epitopes attached to the antibody is necessary. Taken together, our data demonstrated that multiple AECs could sensitize cancer cells to virus-specific T cells.

## Introduction

Antibody-mediated immunotherapies like immune checkpoint blockade (ICB) have demonstrated to be effective for the treatment of tumors by activating the patient’s own immune system (1). Since its efficacy depends on the mutational burden of the tumor and the efficiency of T-cell infiltration, the treatment is not successful in patients suffering from cancers with low immunogenicity (2, 3). Moreover, a large fraction of tumor infiltrating lymphocytes (TILs) is not tumor specific, as a wide range of epitopes presented in HLA class I and II molecules unrelated to the tumor can be recognized (4).

A second class of immunotherapies, are CD3-bispecific antibodies (CD3-BsAbs), which regardless of the specificity of the T-cell receptor (TCR) engages all CD3^+^ T-cells and due to the crosslinking with the cancer cells results in T-cell activation and tumor cell killing (5, 6). However, in contrast to ICB, one of the largest hurdles with CD3-BsAbs is overactivation of the CD3^+^ T-cells, followed by excessive release of inflammatory cytokines, which may trigger the cytokine release syndrome (CRS) (7).

An interesting group of T-cells present among TILs are T-cells specific for viral antigens (8, 9). In particular, exposure to the herpesviruses Epstein Barr-virus (EBV) and Cytomegalovirus (CMV) results in persistent infections in a large fraction of the human population and both viruses induce very potent T-cell responses as a consequence of their occasional reactivations (10). There is a relatively small number of immunodominant T-cell epitopes and in infected individuals high frequencies of EBV- and CMV-specific T-cells can be present (11, 12).

Virus-specific CD4^+^ (13, 14) and CD8^+^ T-cells (15, 16) can be redirected towards viral antigen-negative cancer cells by coupling antigens or T-cell epitopes to tumor-specific antibodies. We refer to antibodies that are able to redirect CD8^+^ T-cells as antibody-epitope conjugates (AECs). After binding of the AECs to the antibody target on the cancer cell, the virus-peptide epitopes are proteolytically released, and presented by HLA class I molecules, allowing recognition by the virus-specific CD8^+^ T-cells.

AECs have shown great promise (15, 16), but more data is needed to be able to optimize their use and to reveal possible limitations. There are many methods to attach drugs, peptides, or other molecules to antibodies as has been shown by the antibody engineering strategies used in the past (17–19). Here, we compared three engineering strategies for generating AECs and presented data on their functionality, antibody target specificity and the proteolytic activity required for peptide release and T-cell activation. We successfully used a chemical and an enzymatic conjugation strategy as well as genetic fusions to create these AECs and compared them on a functional level.

## Materials and methods

### Antibodies and peptides

Cetuximab (CTX) was obtained from Merck (Germany, Darmstadt). Trastuzumab (TRS), all genetically modified antibodies and the AECs were produced at Genmab *via* transient expression in ExpiHEK293 FreeStyle cells as described before (20) and purified by Protein A affinity chromatography. If required, protein aggregates were removed *via* Size Exclusion Chromatography (SEC) to yield protein product with a >95% monomeric content as analyzed on high performance liquid chromatography-SEC (HPLC-SEC). The genetically fused AECs were produced with the following additional amino acid sequence at the C-terminus of the heavy chains: GGSG-LSGRSDNH-YVLDHLIVV, and the antibodies with the sortase A recognition domain (CTX- and TRS-S-His_6_) with: LPTEGG-HHHHHH. All antibodies were stored in phosphate-buffered saline (PBS).

The following antibodies were used for flow cytometry: Goat Anti-human IgG-A488 (Jackson immunoResearch, UK, Cambridgeshire) or -PE (Jackson immunoResearch), Mouse Anti-FLAG-tag-PE (M2) (Abcam, UK, Cambridge), Mouse anti-HLA-A2 (produced inhouse from clone BB7.2 (21)), and Goat anti-mouse-PE (Ebioscience, USA, San Diego). The antibodies used for western blot were Mouse Anti-FLAG-tag (clone M2, Sigma, USA, St. Louis), Mouse anti-His-tag (Qiagen, Germany, Hilden), Goat anti-human-IgG-IRDye800CW (Licor, USA, Lincoln) and/or Donkey anti-mouse-IgG-IRDye680RD (Licor).

Peptides used were produced within the LUMC and synthesized with Fmoc chemistry and the identity was confirmed with mass-spectrometry and are visualized in **Table 1**. All peptides were dissolved in DMSO (Sigma) at 20 mg/mL before usage.

**Table 1:** List of peptides used.

Conjugation strategy:	Peptide sequence:	Epitope:	Protease cleavage site designed for:
***MAL***	Maleimide-PEG11-*LSGRSDNH*-DYKDDDDK	FLAG-tag	uPa, matriptase and legumain
	Maleimide-PEG11-*LSGRSDNH*-YVLDHLIVV	EBV	
***SrtA***	GGGGG-PEG11-*LSGRSDNH*-DYKDDDDK	FLAG-tag	
	GGGGG-PEG11-*LSGRSDNH*-YVLDHLIVV	EBV	
	GGGGG-PEG11-*LSGRSDNH*-NLVPMVATV	CMV	
	GGGGG-PEG11-*VPLSLYSG*-NLVPMVATV		MMP2-7-9
	GGGGG-PEG11-*NSGAFRTY*-NLVPMVATV		uPA and HK2

The protease cleavage site is visualized in cursive and the epitope is underlined.

### Cell lines

HeLa cells were cultured in Dulbecco’s Modified Eagle Medium (DMEM, Gibco, USA, Waltham), 1% Pen/Strep (Gibco), 10% Fetal Calf Serum (FCS, Biowest, France, Nuaillé). The cDNA encoding HLA-A2 was transduced into the HeLa cells with the aid of a pEF1α lentiviral vector. Transduced cells were selected by supplementing the medium with 250 μg/mL zeocin (Invitrogen, USA, Massachusetts, Waltham). Epidermal growth factor receptor (EGFR) and human epidermal growth factor receptor 2 (Her2) knockout cell lines were generated with an expression cassette encoding the guide RNAs (Sigma, clone ID 123759703 and 244226520) in vector plv-u6g-ppb. Cells were simultaneously transfected with the plasmids containing the gRNA and Cas9. The next day, cells were selected with 2 μg/mL puromycin (InvivoGen, USA, San Diego) for 48 hrs. With FACS-based cell sorting the EGFR- and Her2- negative cell populations were enriched, and knockout (KO) clones were selected by limiting dilution. One of the HeLa-A2 Her2 KO clones was transduced with a truncated-Her2 receptor encoding cDNA in the MP71 retroviral vector. Retroviral transductions were performed as described previously (22). Cell cultures were enriched for transduced cells by FACS sorting using an Aria III cell sorter (BD Bioscience, USA, Franklin Lakes). The T-cells used were CMV (NLVPMVATV presented in HLA-A*02:01) or EBV (YVLDHLIVV presented in HLA-A*02:01) specific T-cell clones, or CD8^+^ T-cells derived from peripheral blood mononuclear cells (PBMCs) that were transduced with the virus-specific TCR or, as a control, with a non-specific TCR as described before (23). T-cells were cultured in T-cell medium (TCM); Iscove`s Modified Dulbecco`s Medium (IMDM, Lonza, Switzerland, Basel), 5% FCS, 5% human serum (Sanquin, the Netherlands, Amsterdam), 3 mM L-glutamine (Lonza), 1% Pen/Strep, and 200 IU/ml IL-2, and stimulated every 10-16 days with phytohaemagglutinin (PHA, ThermoFisher, Germany, Dreieich) and irradiated feeder cells. Before the T-cells were used in the assays they were washed 3 times with IMDM supplemented with 0.5% human albumin (Albuman, Sanquin) to remove expansion-related cytokines.

### Conjugation strategies and purification

For maleimide conjugation, 10 µM wildtype antibody, 500 µM tris(2-carboxyethyl)phosphine (TCEP, Sigma) and 200 µM peptide were added together in PBS, incubated overnight at 22°C whilst shaking. Conjugates were purified over Size Exclusion Chromatography (SEC) on a Superdex200 column (GE Healthcare, Germany, Freiburg) equilibrated in PBS to remove excess peptides. For the sortase A conjugation 25 µM sortase A (produced in house), 5 µM Ab-S-his_6_ with a sortase A recognition motif and a His-tag (LPTEGG-His_6_) on the C-terminus of the heavy chain, and 500 µM peptide were incubated for 45 min at 37 ˚C in sortase A buffer (150 mM NaCl, 50 mM Tris.Cl, 10 mM CaCl2, pH=7.5). The reaction mixture was directly applied to a Superdex 200 SEC column coupled to a His-trap column (GE Healthcare) equilibrated in 100 mM Tris with 20 mM Imidazole to remove unconjugated antibody and His-tagged sortase A enzyme, and conjugate-containing fractions were concentrated on an Amicon concentration filter (MWCO 50K, Merck).

### Analysis of conjugation

The conjugates were analyzed by sodium dodecyl sulfate–polyacrylamide gel electrophoresis (SDS-PAGE) on hand-casted gels (12,5%) in Laemmli sample buffer under reducing (in the presence of 20 mM Dithiothreitol (DTT)) or non-reducing conditions (no DTT added). The gels were either stained with an instant blue staining (Abcam) or were subjected to western blot analysis. For western blots, the gels were transferred onto nitrocellulose membranes (Biorad, Germany, Feldkirchen) with the Trans-Blot Turbo Transfer system (Biorad). Membranes were blocked with 5% milk dissolved in PBS and stained with primary antibodies diluted in 1% milk in PBS, containing 0.05% Tween 20 (PBST). Next, the membranes were washed 2x with PBST and incubated with secondary antibodies in 1% milk in PBST. to visualize the primary antibodies on the Odyssey DLx (Licor). The epitope-to-antibody ratio`s (EARs) were estimated or determined by Hydrophobic Interaction Chromatography (HIC), SDS-PAGE or intact liquid chromatography-mass spectrometry (LC-MS). Estimations on SDS-PAGE were made with Image Studio Lite (version 5.2) or Fuji Image J.

### Flow cytometry analysis

Cells were plated in a 96-wells U bottom plate and washed with PBS, containing 0.5% bovine serum albumin (BSA) and 0.02% sodium azide (PBA). Cells were incubated with primary antibody for 30 minutes on ice. Hereafter, the cells were washed with PBA, followed with 20 minutes incubation with the secondary antibody. The cells were washed once more and analyzed with fluorescence-activated cell sorting (FACS) on a LSRII (BD Bioscience).

### T-cell activation and killing

To determine T-cell activation, 5,000 target cells/well were seeded in a 384-well flat-bottom tissue culture plate and incubated overnight to let them adhere. Antibody titrations were prepared in IMDM supplemented with 0.5% human albumin, and the target cells were incubated for 1 hour at 37 ˚C with the antibodies. The antibody solution was thoroughly washed away by 3 wash steps with IMDM supplemented with 0.5% human albumin and 4,000 T cells/well were added to the target cells in IMDM supplemented with 0.5% human albumin and 200 IU/ml IL-2. After an overnight incubation, part of the supernatant was harvested and the interferon-y (IFN-y) production was assayed by enzyme linked immune sorbent assay (ELISA) as a marker for activation (Diaclone, France, Besancon) according to manufacturer’s protocol.

To investigate the killing potential of the T-cells, T-cell medium was added and the plates were incubated for another 48 hours. Subsequently, T-cells were removed by washing 3 times with IMDM supplemented with 0.5% human albumin, followed by the addition of HeLa culturing medium supplemented with 10% AlamarBlue HS cell viability reagent (ThermoFisher). Viability was measured according to the manufacturer’s protocol. The percentage target cell killing was calculated using:


$$\%killing=100-\frac{\left(RFU_{sample}-averageRFU_{background}\right)}{\left(RFU_{no\;addition}-averageRFU_{background}\right)}\times 100$$

In which RFU_sample_ is the measured relative fluorescent units (RFU) of the sample, averageRFU_background_ represents the RFU that comes from wells in which only T-cells were cultured and RFU_no addition_ represents the RFU of a coculture of target and effector T cells which did not receive any antibody or peptide treatment.

### Acidic heat treatment and aggregation assay

Antibody binding was diminished by incubating the antibodies at a concentration of 3.75 mg/ml (25 µM) at 98°C for 1 hour with 0.2 M HCl. Before the antibody was taken along in a T-cell assay it was diluted to a concentration of 80 nM in IMDM after which the pH was verified by looking at the phenol red indicator present in the medium. The aggregation between the different batches was measured with the PROTEAOSTAT protein aggregation assay according to manufacturer’s protocol (Enzo Life Sciences, Germany, Lörrach).

### Statistical analysis

Graphpad Prism software (V.9.0.1) was used to perform the statistical analysis. In the legend of the figure the used test is indicated. The significance levels are indicated as ns: not significant, *p<0.05, **p<0.01, and ***p<0.001.

### Ethics approval

This study involves materials from human participants and was approved by Institutional Review Board of the Leiden University Medical Center (approval number 3.4205/010/FB/jr) and the METC-LDD (approval number HEM 008/SH/sh). Materials from healthy individuals were collected after written informed consent.

## Results

### Three conjugation strategies to attach epitopes to antibodies

**Figure 1:**
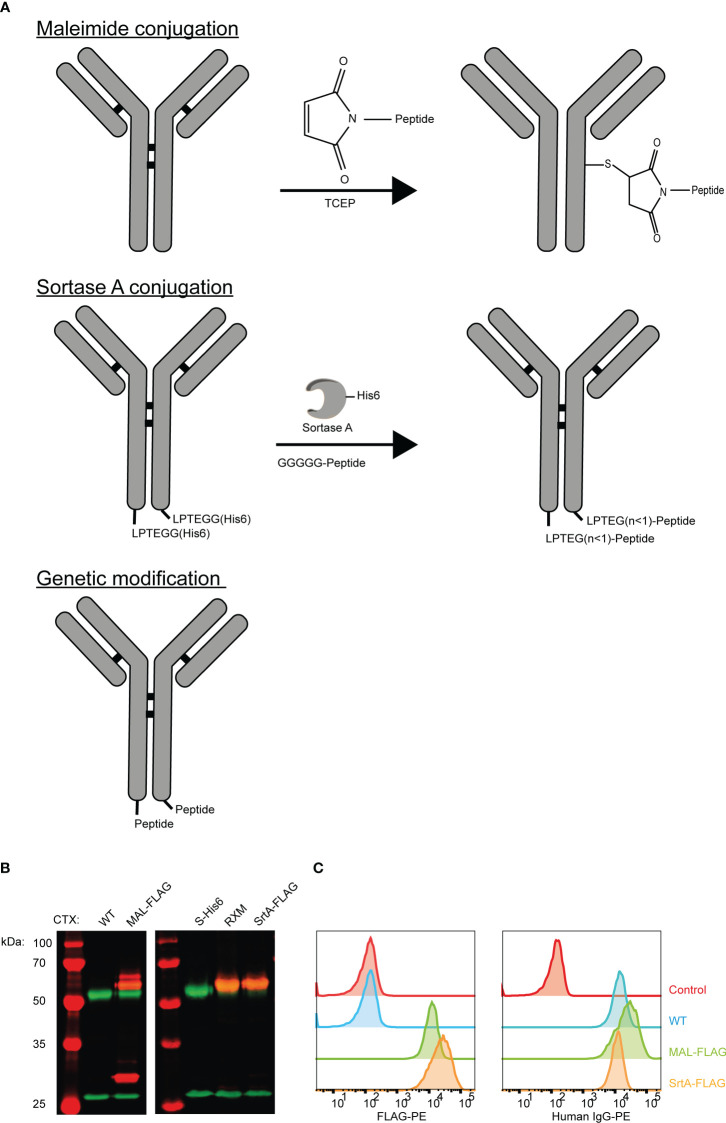
Three strategies to generate AECs: chemical, enzymatical and genetic. **(A)** Schematic representation of the reaction and/or the end-product of the 3 conjugation strategies: maleimide conjugation, Sortase A conjugation, and genetic modification. For the maleimide conjugation an example with 1 epitope is given after reduction of the disulfide bridges. **(B)** Analysis of the FLAG-tag epitope conjugated to CTX by Western blot with in red the molecular weight marker and FLAG-tag signal, and in green the human IgG signal. For CTX-SrtA-FLAG, the parental antibody with sortase recognition domain (S-His6) and the reaction mixture (RXM) are shown. **(C)** FACs analysis to visualize the binding of the CTX conjugates with FLAG-tag, to HeLa cells expressing EGFR. As a control a single anti-human-IgG-PE or anti-FLAG-PE staining was taken along.

Three approaches were applied to create AECs: chemical conjugation (maleimide reaction), enzymatic conjugation (sortase A conjugation), and genetic fusion (**Figure 1A**). The maleimide and sortase A (SrtA) conjugation reactions were optimized using a FLAG-tag containing peptide on cetuximab (CTX). CTX binds to the epidermal growth factor receptor (EGFR) that is overexpressed by many different cancerous cells. For the maleimide conjugation reaction the four disulfide bridges were reduced with a reducing agent to allow the maleimide to react with the free thiols, which results in a heterogeneous conjugation mixture with an epitope-to-antibody ratio that can range from 0-8. For the SrtA reaction, CTX was produced with a SrtA recognition site (LPTEG) on the C-terminus of the Heavy Chain (HC) followed by a His_6_-tag (CTX-S-His_6_). With maleimide and SrtA conjugation, FLAG-tag containing CTX conjugates were generated (CTX-MAL-FLAG and CTX-SrtA-FLAG, respectively) and excess peptide was removed by SEC (**Figure S1A**). For the SrtA conjugation an additional step was taken to remove SrtA and unconjugated CTX-S-His_6_ from the reaction mixture (RMX) with a His-trap column (**Figure S1B**).

To determine if conjugation was successful, conjugates were analyzed by Western blot (**Figure 1B**). For CTX-MAL-FLAG two strong bands of FLAG-tagged protein appeared above the HC of CTX, which correspond to 1 or 2 conjugations on the HC and one FLAG-tagged band appeared above the light chain (LC), indicating that the disulfide bridges are reduced and conjugation has taken place. However, there was still a fraction of unconjugated CTX present as approximately 50% of the HC did not contain a FLAG-tag. For CTX-SrtA-FLAG, only one Flag-tagged band with a higher molecular weight than the unconjugated HC was observed, indicating that conjugation only took place at the SrtA recognition site.

We further analyzed the conjugation of the FLAG-tag on CTX and the capability of CTX to retain binding to its receptor after conjugation by flow cytometry. Both conjugates bound to the EGFR positive HeLa cells, and the FLAG-tag was detected on both conjugates, showing that the conjugation did not affect antibody target binding and that the FLAG-tag was attached to the antibody (**Figure 1C**). Since the long-term stability of a maleimide-thiol linker cannot be guaranteed as it can be converted back to the starting thiol and maleimide (**Figure S2**) (24), all purified antibody conjugates were stored at -80°C, which kept the conjugates stable over a longer period of time. By using the FLAG-tagged peptides, we confirmed that both the MAL and SrtA conjugation strategy could be used to attach peptides.

### Conjugation of an EBV CD8^+^ T-cell epitope to tumor-targeting antibodies

Next, we aimed to conjugate the highly immunogenic EBV BRLF1/A2 T-cell epitope to CTX using the MAL and SrtA strategies and generated the genetic variant (CTX-GEN) for comparison. We chose the EBV BRLF1/A2 epitope based on the high frequencies of BRLF1-reactive T-cells in EBV-positive individuals and the lack of a cysteine in the sequence as free thiols are not compatible with maleimide conjugation.

**Figure 2:**
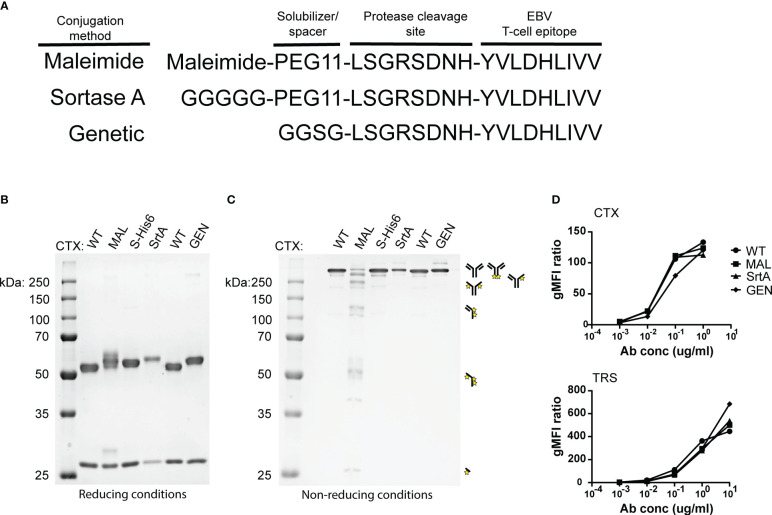
An EBV T-cell epitope preceded by a protease cleavage sequence can be conjugated chemically, enzymatically, or genetically to tumour-specific antibodies. **(A)** The amino acid sequences of the peptides that are used in the reaction for maleimide- and sortase A conjugation and the peptide sequence that is attached genetically to CTX. The three conjugation strategies analysed by SDS-PAGE visualized with an Instant Blue staining **(B)** under reducing and **(C)** non-reducing conditions. With the conjugation possibilities for CTX-MAL depicted next to the gel. The conjugation possibilities were determined by looking at the most abundant bands. **(D)** To determine whether the antibody conjugates still bind their target, titrations were performed with CTX antibody conjugates on HeLa-A2 cells and with TRS antibody conjugates on HeLa-A2 Her2 cells and binding potential was analysed by flow cytometry. Bound antibody conjugates were visualized with anti-human IgG-PE and representative data of two independent experiments are shown.

To allow release of the EBV epitope, the protease cleavage site LSGRSDNH that is specific for urokinase-type plasminogen activator (uPA), membrane-type serine protease 1 (MT-SP1/matriptase), and legumain (25) was included at the N-terminus of the EBV T-cell epitope. For MAL and SrtA conjugations, a poly(ethylene glycol)-11 (PEG11) linker was introduced at the cleavage site to make the peptide more hydrophilic and increase the conjugation levels and yields. For the genetically fused antibodies a small spacer (GGSG) was placed in between the antibody and the protease cleavage site (**Figure 2A**).

The three differently generated AECs were analyzed on SDS-PAGE under reducing (**Figure 2B**) and non-reducing conditions (**Figure 2C**). Maleimide conjugation yields heterogenic product under reducing conditions, as additional bands appeared for CTX-MAL above both HC and LC. Contrary to this and as expected, homogeneous product was obtained for CTX-SrtA and -GEN where a single HC band is observed at a higher apparent molecular weight. Under non-reducing conditions we observed the predicted antibody fragmentation pattern for CTX-MAL, as cysteine conjugation impairs formation of oxidized disulfide bridges. We also conjugated the EBV BRLF1/A2 epitope to an alternative antibody, trastuzumab (TRS), and similar results were obtained as for CTX (**Figure S3**). TRS binds the human epidermal growth factor receptor 2 (Her2) and, similar to CTX, is overexpressed in many cancers, primarily in breast and ovarian carcinomas. The epitope-to-antibody ratio (EAR) was estimated with Hydrophobic Interaction Chromatography (HIC), mass-spectrometry (**Figure S4**), or SDS-PAGE, and summarized in **Table S1** and showed that all three conjugation strategies resulted in an EAR of at least 2.

As the attached peptides are very hydrophobic and in the case of MAL conjugation also tertiary structure changes in the antibody can occur, the receptor-binding properties of the AECs were analyzed by flow cytometry. All AECs were titrated on HeLa-A2 cells and binding efficiency was compared to the wildtype antibody (WT) (**Figure 2D**). Since, HeLa cells only express Her2 at low levels, the TRS-AECs were tested on HeLa-A2 cells transduced with a truncated Her2, which lacks the intracellular domain (HeLa-A2 tHer2). No significant differences were observed in target binding capacity between the differently generated AECs compared to either CTX-WT or TRS-WT. These results demonstrate that AECs can be generated using these three different methods without changing the binding properties of the antibodies.

### AECs sensitize tumor cells to be recognized by EBV-specific T cells

**Figure 3:**
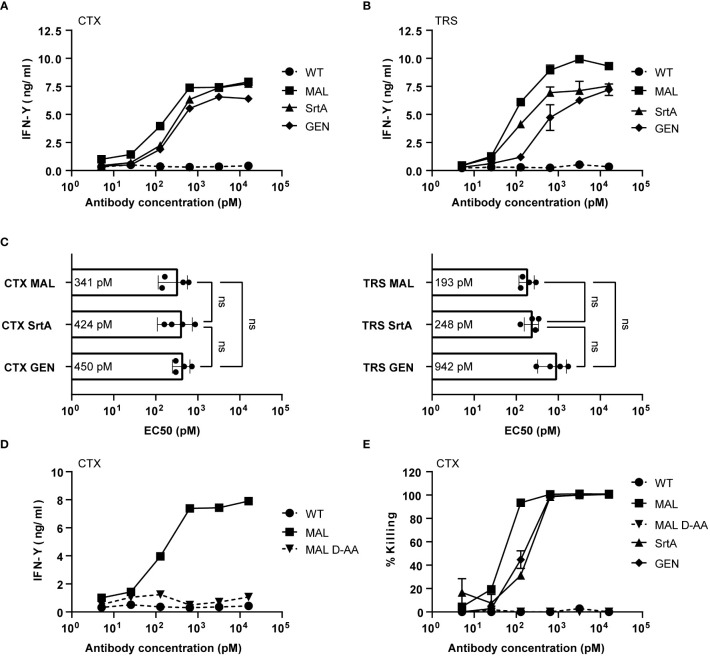
EBV-AECs can sensitize tumour cell lines to be recognized and killed by EBV-specific T cells, irrespective of the strategy used for conjugation. CTX-MAL, -SrtA and -GEN AECs **(A)** and TRS-MAL, -SrtA and -GEN AECs **(B)** were titrated on HeLa cells transduced with HLA-A2 (HeLa-A2) for CTX-AECs and on HeLa-A2 tHer2 for TRS-AECs and incubated overnight with EBV-BRFL1/A2-specific T-cells. Supernatant was harvested and IFN-y production as measure for T-cell activation was analysed by IFN-y ELISA. In **(C)** the EC_50_s of the different AECs were calculated and plotted. For the statistical analysis, a RM one-way ANOVA with Tukey`s multiple comparisons was performed. **(D)** To test whether proteolytic activity is necessary CTX-MAL and CTX-MAL_D-AA_ were taken along in a coculture assay and T-cell activation was analysed by IFN-y ELISA. **(E)** To check for target cell killing, an AlamarBlue assay was performed. **(A, B, D, E)** Plotted values are means of duplicates (SEM) and graphs shown are representative figures of an n>3.

To determine whether the different AECs demonstrate potent T-cell activation, HeLa-A2 cells were incubated for 60 minutes with the different CTX-AECs, washed extensively, and co-cultured for 18 hours with EBV BRLF1/A2 specific T-cells (**Figure 3A**). Interferon-γ (IFN- γ) concentration in the culture supernatant was determined as measure for T-cell activation. All three CTX-AECs were able to efficiently activate the T-cells at low antibody concentrations. TRS-AECs incubated with HeLa-A2 tHer2 showed similar results (**Figure 3B**). All AECs had a half maximum effective concentration (EC_50_) in the range of 0.2-1 nM (**Figure 3C**), with no significant difference between the three different conjugation strategies for both CTX- and TRS-AECs. To show that the T-cell activation is specific for recognition of the EBV BRLF1/A2 epitope, the cocultures with the AECs were repeated with a CMV pp65/A2 specific T-cell line. The CMV pp65/A2 T-cells only showed T-cell activation when incubated with the pp65/A2 peptide (**Figure S5**).

To confirm that proteolytic processing and release of the EBV T-cell epitope is required for T-cell activation a non-cleavable peptide was conjugated to CTX. Amino acids in the proteolytic cleavage site of the conjugated peptide used in the maleimide conjugation were substituted by amino acids in the D conformation (MAL_D-AA_), which reduce recognition by the proteases (26). The CTX-MAL and CTX-MAL_D-AA_ conjugates have a similar EAR (**Figure S4**) and therefore could be compared in biological assays. Results shown in **Figure 3D** clearly demonstrate that D- amino acid substitution completely prevent processing and therefore T-cell activation. To determine whether the AECs were able to sensitize target positive tumor cells to be killed by EBV-reactive T cells an AlamarBlue viability assay was performed. Interestingly, all three CTX-AECs efficiently induced high HeLa-A2 target cell killing by the EBV-reactive T cells, whereas no killing was observed when HeLa-A2 cells were incubated with CTX-WT or CTX-MAL_D-AA_ (**Figure 3E**). This was also observed for HeLa-A2 tHer2 cells incubated with the different TRS-AECs (**Figure S6**).These results clearly demonstrate that all three conjugation methods are suitable and that proteolytic processing is required to induce efficient T-cell activation and tumor-cell killing by the T-cells at sub-nanomolar antibody concentrations.

### Cell surface expression of antibody target is essential for T-cell epitope delivery

**Figure 4:**
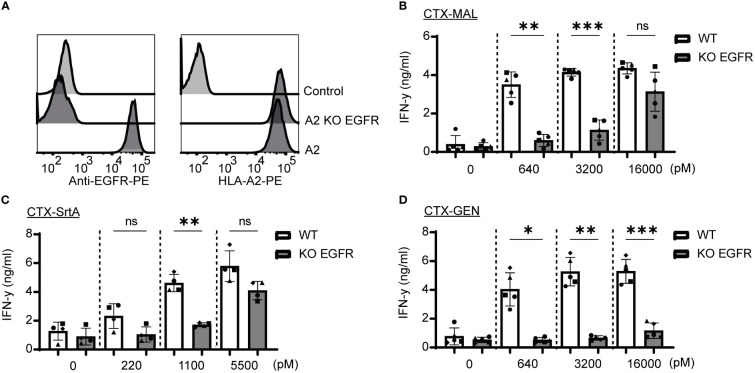
Recognition and activation of the T-cells is target specific and shows differences at high concentrations. **(A)** For both targets KO cells were generated by using CRISPR/Cas9 technology and analysed with FACS for EGFR and HLA-A2 expression **(B–D)** The three CTX conjugates were also titrated on the HeLa-A2 KO EGFR alongside the HeLa-A2 cells with wildtype (wt) EGFR expression for the three highest concentrations and T-cell activation was measured with an IFN-y ELISA. Plotted values are the means of duplicates within one experiment of at least three independently performed experiments. For the statistical analysis, a repeated measurements one-way ANOVA with Šidák multiple comparisons was performed.

To determine whether the release of the EBV T-cell epitope from the AECs was target specific, an EGFR knockout (KO) HeLa-A2 cell line was generated (HeLa-A2 EGFR KO) (**Figure 4A**). Incubation of the different CTX-AECs with HeLa-A2 cells, either EGFR WT or KO, co-cultured for 18 hours with EBV-reactive T cell demonstrated a significant difference in activation of the T-cells. EBV T-cell activation only occurred when EGFR was expressed by HeLa-A2 cells, indicating that AEC binding to target positive tumor cells facilitates cleavage of EBV peptide and binding to HLA-A2 expressed by the tumor cells. At the highest AEC concentration, no significant difference in IFN-γ production by the EBV-T cells was observed when CTX-MAL and CTX-SrtA were incubated with EGFR-positive and -negative HeLa-A2 cells (**Figures 4B, C**). This target independent activation of T cells at the highest concentration of AECs was not observed for CTX-GEN (**Figure 4D**) and TRS-GEN (**Figure S7**).

Next, we determined whether the stability of the AECs could explain the target-independent activation of EBV-T cells observed on the EGFR KO cells when incubated with high concentrations of CTX-MAL and CTX-SrtA. CTX-GEN was treated with an acidic heat shock (CTX-GEN_AHT._) to induce denaturation and therefore its ability to bind to EGFR (**Figure S8A**) and repeated the cocultures (**Figure S8B**). Low amounts of denatured antibodies already resulted in a target independent recognition of EGFR KO cell lines (**Figure S8C**), suggesting that a reduced stability might cause the target independent T-cell activation at the highest concentrations. As (partial) unfolding of the antibody is also linked to aggregation, the conjugates stored at -80°C were checked for aggregation with a fluorescence-based aggregation assay (**Figure S8D**), which showed that no aggregates were present at the start of our assays. In conclusion, these experiments show that denatured antibodies can activate T-cells independent of the antibody target. However, the delivery of the EBV epitope by stable AEC is target specific.

### Screening of protease cleavage site/epitope combinations

**Figure 5:**
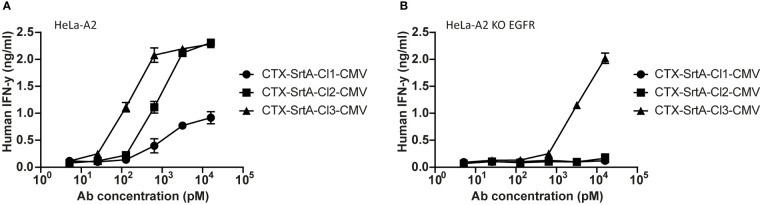
The CMV epitope NLV/A2 epitope was conjugated with sortase A with three different cleavage sites. **(A)** These conjugates were titrated on HeLa-A2 **(B)** or HeLa-A2 EGFR KO cells and incubated with CMV NLV/A2-specific T-cells. T-cell activation was measured with an IFN-y ELISA. Plotted values are the means of duplicates (+/- SEM) and graphs shown are representative figures of an n=3.

To demonstrate the modularity of the designed approach and explore if the patient target group could for instance be expanded, in the next set of experiments we tested the conjugation of another immunodominant epitope, the CMV pp65/A2 epitope. We chose the SrtA conjugation method for further screening purposes. This method is relatively quick and results in a homogeneous end-product with 2 epitopes on the C-terminus of the HC, allowing for straightforward comparison of the efficiency of different cleavage sites. The CMV pp65/A2 epitope was synthesized with three different proteolytic cleavage sites: the same cleavage site as used for the EBV BRLF1/A2 epitope (CTX-SrtA-cl1-CMV), one for matrix metalloproteinase (MMP)2, 7 and 9 (CTX-SrtA-cl2-CMV), and one for human kallikrein-related peptidase 2 (HK2) and uPa (CTX-SrtA-cl3-CMV) and conjugated with the sortase A conjugation method to CTX. CTX-SrtA-cl1-CMV did not efficiently activate T-cells as previously shown for the EBV-T cells, whilst the other two AECs showed T-cell activation at concentrations of 0.1-1 nM (**Figure 5A**).

Notably, when tested on the EGFR KO cell line, CTX-SrtA-cl3-CMV showed target-independent T-cell activation (**Figure 5B**). Even though there was still a 30-fold difference between target-dependent and target-independent T-cell activation observed, CTX-SrtA-cl2-CMV would be the preferred choice. These results suggest that the peptide/protease cleavage site attached can induce differences in the stability of the AECs. Moreover, it demonstrates that it is possible to generate AECs with another immunodominant epitope and that the cleavage site in front of the CMV pp65/A2 epitope influences the efficiency.

## Discussion

The first studies with antibody-epitope conjugates (AECs) demonstrated that these AECs are promising new modalities to deliver immunogenic epitopes and redirect virus-specific T-cell activity towards cancer cells (13, 15, 16, 27), tackling hurdles such as lack of immunogenicity of tumors and possibly also CRS (28, 29). Here we describe the successful generation of AECs using three conjugation methods (chemical and enzymatical conjugation and genetic fusion).All conjugation methods demonstrated to be able to efficiently redirect EBV virus-specific CD8+ T-cell reactivity towards tumor cells and showed an antibody target dependency on target KO cell lines. The KO cell lines proved to be a valuable tool to look at specificity and therefore at the stability. The genetic AECs exhibited the highest stability and therefore have our preference for future clinical studies, while maleimide and SrtA conjugation may be more useful for defining the ideal antibody target, viral epitope, as well as epitope-protease cleavage-site combination or the mechanism of action.

All three methods can be used to gain a better insight into the field of AECs. Depending on the question asked and the materials at hand, the most suitable method can be selected. For example, to screen different antibody targets, maleimide conjugation offers the opportunity to test existing monoclonal antibodies as genetic modification is not necessary. Whilst for the comparison of different protease cleavage site-epitope combinations, SrtA or direct genetic fusion are the preferred choices as they result in a defined stoichiometry of epitopes and facilitate screening and comparing of the different combinations with the same antibody target. For future purposes, multiple protease cleavage site-epitope combinations should be screened to test which combination has the highest efficiency, but also to see if the concept can be broadened with additional epitopes. Next to that, a 2-step conjugation approach like maleimide and SrtA conjugation, gives the ability to include unnatural amino acids in the peptide’s sequences such as D-amino acids, citrullinated amino acids, or other modifications such as fluorophores (30–32). This may increase the possibilities or could help elucidate the mechanism-of-action, whilst this would not be possible with a more direct approach like genetic fusions. It should be noted, though, that in the case of maleimide conjugation, cysteine-containing protease cleavage sites and epitopes (e.g., the EBV LMP-2/A2 ( **C** LGGLLTMV) and the BMLF-1/A2 (GL **C** TVAML) epitopes) cannot be used as that could result in unwanted cross-linking *via* disulfide bridge formation (12, 33).

On the other hand, one of the advantages of the direct genetic approach is that the obtained conjugates are well-defined and can be easily characterized and quantified with LC-MS, which can be more complicated for the AECs generated with maleimide chemistry and SrtA as these contain a PEG linker (34, 35). Removal of the PEG11 is possible, however in the case of SrtA conjugation the yield will drop drastically. Moreover, the SrtA conjugates are homogenous in their number of epitopes, but not necessarily in the amount of glycine residues attached between the antibody and the epitope (36) (**Figure 1A**).

For research purposes all three methods have their merits. However, for future therapeutic application aspects such as stability and aggregation propensity, which is correlated to *in vivo* half-life and/or immunogenicity are of a crucial importance (37). In addition, aggregation is evidently unwanted as it was already a challenge in the clinic and could reduce the efficiency, cause anti-drug antibodies (ADAs), and could result in a low production yield (38–40). We observed for two monoclonal antibodies with different targets that the conjugation strategy, but also the attached peptide, affects the stability of the AECs (35, 41). This also indicates that further testing and screening is necessary to find the AECs not only with the highest efficiency, but also the highest stability. The AECs can potentially avoid two of the main hurdles observed for ICB and CD3-BsAbs. It has previously been shown that the intratumoral delivery of virus epitopes can promote a long-term antitumor immune response on top of the virus-mediated immune response (42) and by reducing the number of T-cells that can be triggered the chances of CRS mediated by CD3 binding can be reduced. Moreover, it should not be forgotten that the AEC concept can be applied to already existing monoclonal antibodies, which potentially on top of the redirection of the virus-specific T-cells allows for antibody mediated effects and a functional Fc-backbone (43).

In conclusion, our data demonstrates that AECs can be efficiently generated with three different conjugation strategies. All three AECs induced T-cell activation and tumor cell killing, which was mediated by proteolytic release of the epitope, and was dependent on the presence of the antibody target for all stable AECs. For screening and other research purposes the SrtA conjugation method is preferred as the method is fast and results in homogeneous product with a consistent EAR. Screening of multiple combinations is necessary to give insight in stability and efficiency in which antibody target KO cell lines proved to be an important control. However, our preferred method for further in-depth studies to attach epitopes to antibodies would be genetically fused, as it is well-defined and showed to have the highest stability. This technology may aid further clinical development of antibody-epitope conjugates that can sensitize cancer cells to pre-existing virus-specific T cells.

## Data availability statement

The raw data supporting the conclusions of this article will be made available by the authors, without undue reservation.

## Author contributions

WvdW, BB, JD, PP, RGH, SvK, JS, MR, and MH, designed the research and analyzed results. WvdW, AG, BB, RSH, CA, and RK performed the experiments. WvdW, RCH, MR, and MH wrote the first draft of the paper. All authors contributed to the article and approved the submitted version.

## Supplementary material

Supplementary Figure 1 Homogeneous CTX-SrtA purified by SEC followed by a His-trap column. Samples were loaded in the following order: the unconjugated parental antibody (S-His_6_), the reaction mixture of CTX-SrtA-FLAG before purification (RXM) and after purification (SrtA-FLAG). The Western blot was stained with both anti-His-tag (HIS) antibody and anti-human IgG (Ab).

Supplementary Figure 2 Long-term stability of CTX-MAL conjugates cannot be guaranteed as they can be converted back to the starting thiol and maleimide. **(A)** CTX-MAL-FLAG conjugates were kept at 4°C for different periods of time, before being analysed by Western blot. **(B)** Quantification of the signal on the HC and LC of the anti-FLAG (FLAG) and anti-human IgG (Ab) measured by Western blot was quantified and the % of residual FLAG-tag epitope was calculated for HC and LC combined and separately.

Supplementary Figure 3 An EBV epitope preceded by a protease cleavage sequence can be conjugated chemically, enzymatically, or genetically to TRS. **(A)** All three the different conjugation strategies analysed with SDS-PAGE visualized with an instant blue staining under reducing and **(B)** non-reducing conditions. With the conjugation possibilities for CTX-MAL depicted next to the gel. The conjugation possibilities were determined by looking at the most abundant bands.

Supplementary Figure 4 Mass-spec and HIC analysis. **(A)** HIC analysis of the CTX-MAL conjugates and **(B)** the intact Mass-spec analysis of the CTX-GEN.

Supplementary Figure 5 The CTX- and TRS-AECs are not recognized by a non-specific CMV pp65/A2 specific T-cell line . HeLa-A2 ther2 cells were incubated with 16 nM of the different AECs, followed by a coculture with a CMV pp65/A2 specific T-cell line. T-cell activation was measured with an IFN-y ELISA. As a positive control, cells were incubated with the pp65/A2 peptide.

Supplementary Figure 6 Target cell killing is also observed for HeLa-A2 tHer2 incubated with the different TRS-AECs. To check for target cell killing, an AlamarBlue assay was performed. Plotted values are means of duplicates (SEM) and the shown graph is a representative figure of an n>3.

Supplementary Figure 7 Recognition and activation by the T-cells is target specific but show at high concentration differences between the conjugation strategies also for Trastuzumab. **(A)** KO cells were generated by using CRISPR/Cas9 and analysed with FACS for Her2 and HLA-A2 expression **(B-D)** All three TRS conjugates were titrated on the HeLa KO Her2 alongside the HeLa-A2 tHer2 and T-cell activation was measured with an IFN-y ELISA. Plotted values are the means of duplicates within one experiment of at least three independently performed experiments. For the statistical analysis a RM one-way ANOVA with Sidak multiple comparisons was performed.

Supplementary Figure 8 Denatured AECs can result in T-cell activation independent of the antibody target. **(A)** CTX-GEN and CTX-GEN_AHT_ were titrated in an FACS experiment to check whether the treated antibodies were still able to bind **(B)** CTX-GEN and CTX-GEN_AHT_ were incubated on HeLa-A2 (target positive) and HeLa-A2 EGFR KO (target negative) cell lines. **(C)** CTX-GEN was mixed with CTX-GEN_AHT_ in different ratios (v/v) and incubated on the HeLa-A2 EGFR KO cell line. **(D)** Antibody aggregation was measured for the TRS conjugates with the PROTEOSTAT protein aggregation assay, in which the positive control is a Sortase A conjugate that is already known to aggregate. Plotted values are the means of duplicates within one experiment of at least three independently performed experiments.
