# Supplementary figures and images for: Comparison of methods generating antibody-epitope conjugates for targeting cancer with virus-specific T cells
Source: Front Immunol. 2023 May 16;14:1183914. doi: 10.3389/fimmu.2023.1183914 (PMC10227578; doi:10.3389/fimmu.2023.1183914)

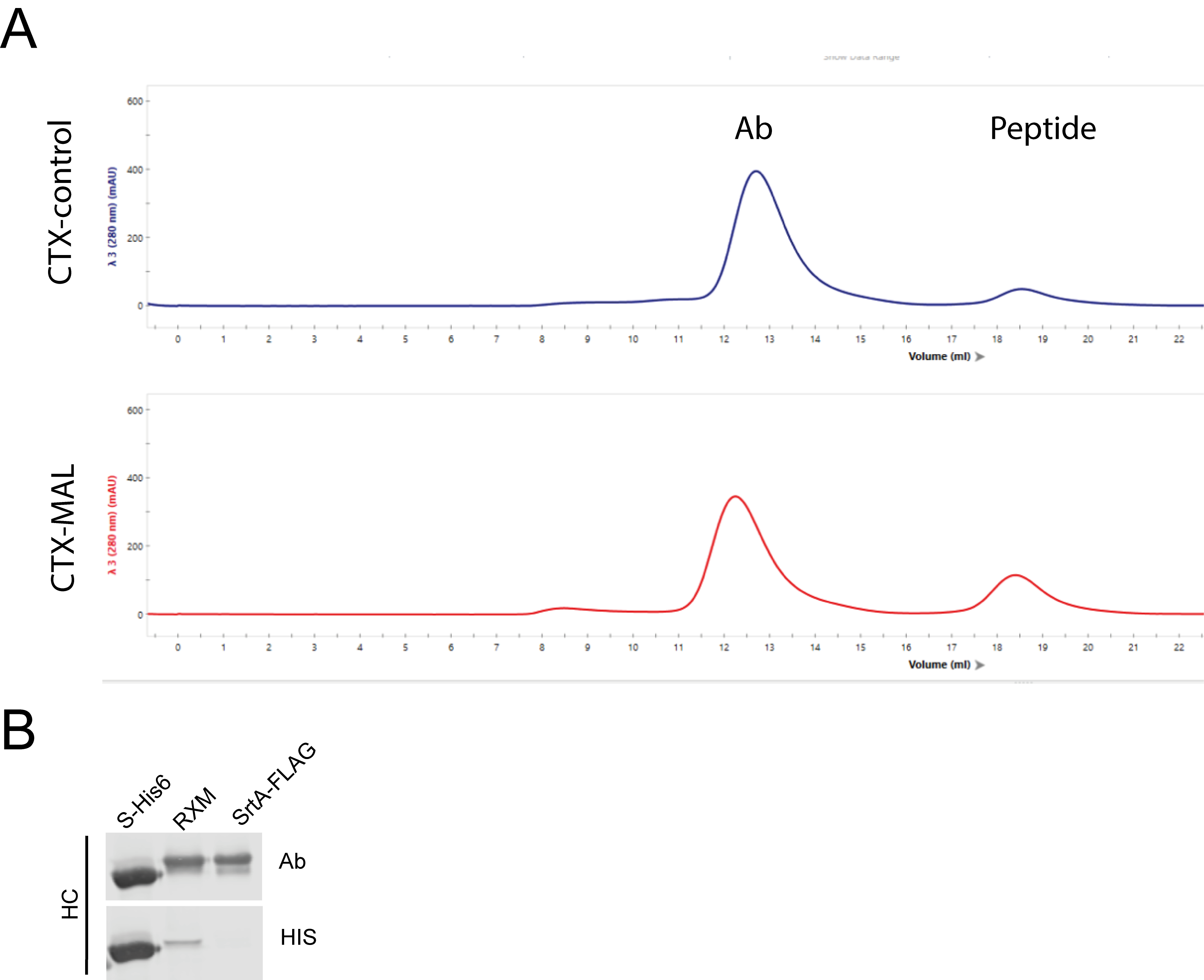

Supplement: Supplementary Figure 1 — Homogeneous CTX-SrtA purified by SEC followed by a His-trap column. Samples were loaded in the following order: the unconjugated parental antibody (S-His6), the reaction mixture of CTX-SrtA-FLAG before purification (RXM) and after purification (SrtA-FLAG). The Western blot was stained with both anti-His-tag (HIS) antibody and anti-human IgG (Ab). [file Image_1.tif]

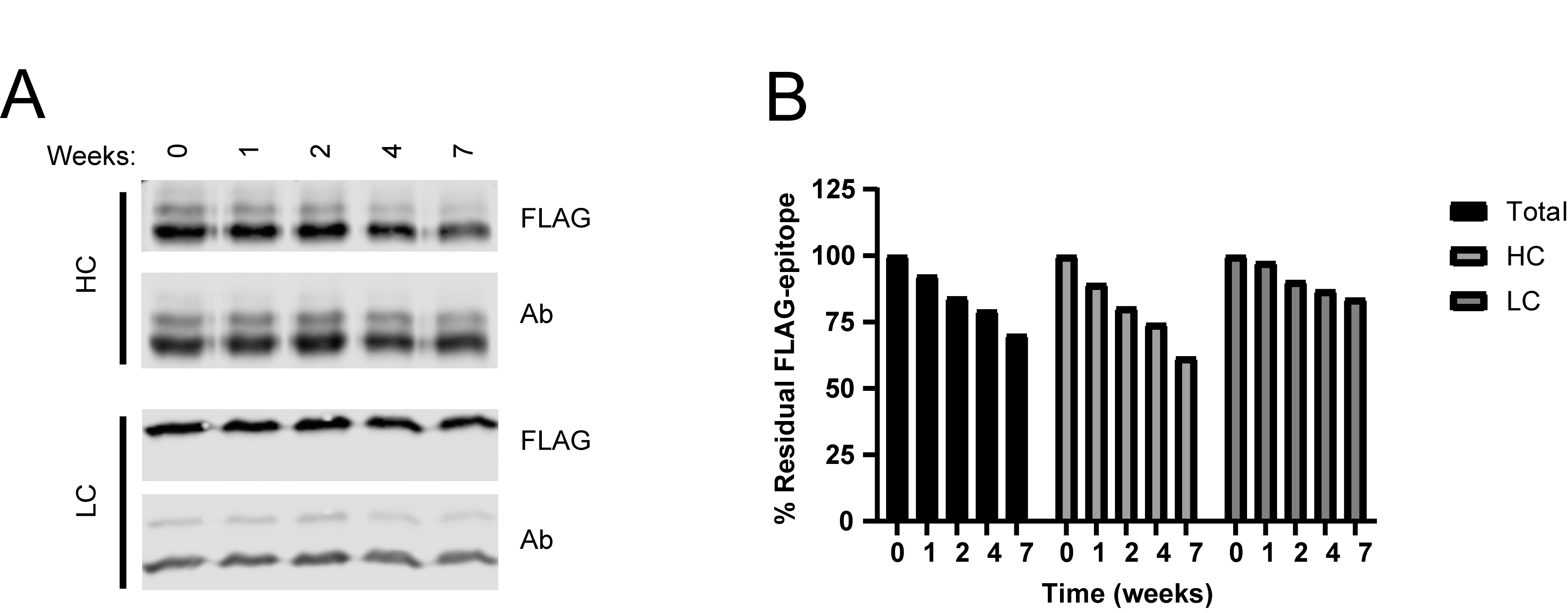

Supplement: Supplementary Figure 2 — Long-term stability of CTX-MAL conjugates cannot be guaranteed as they can be converted back to the starting thiol and maleimide. (A) CTX-MAL-FLAG conjugates were kept at 4°C for different periods of time, before being analysed by Western blot. (B) Quantification of the signal on the HC and LC of the anti-FLAG (FLAG) and anti-human IgG (Ab) measured by Western blot was quantified and the % of residual FLAG-tag epitope was calculated for HC and LC combined and separately. [file Image_2.tif]

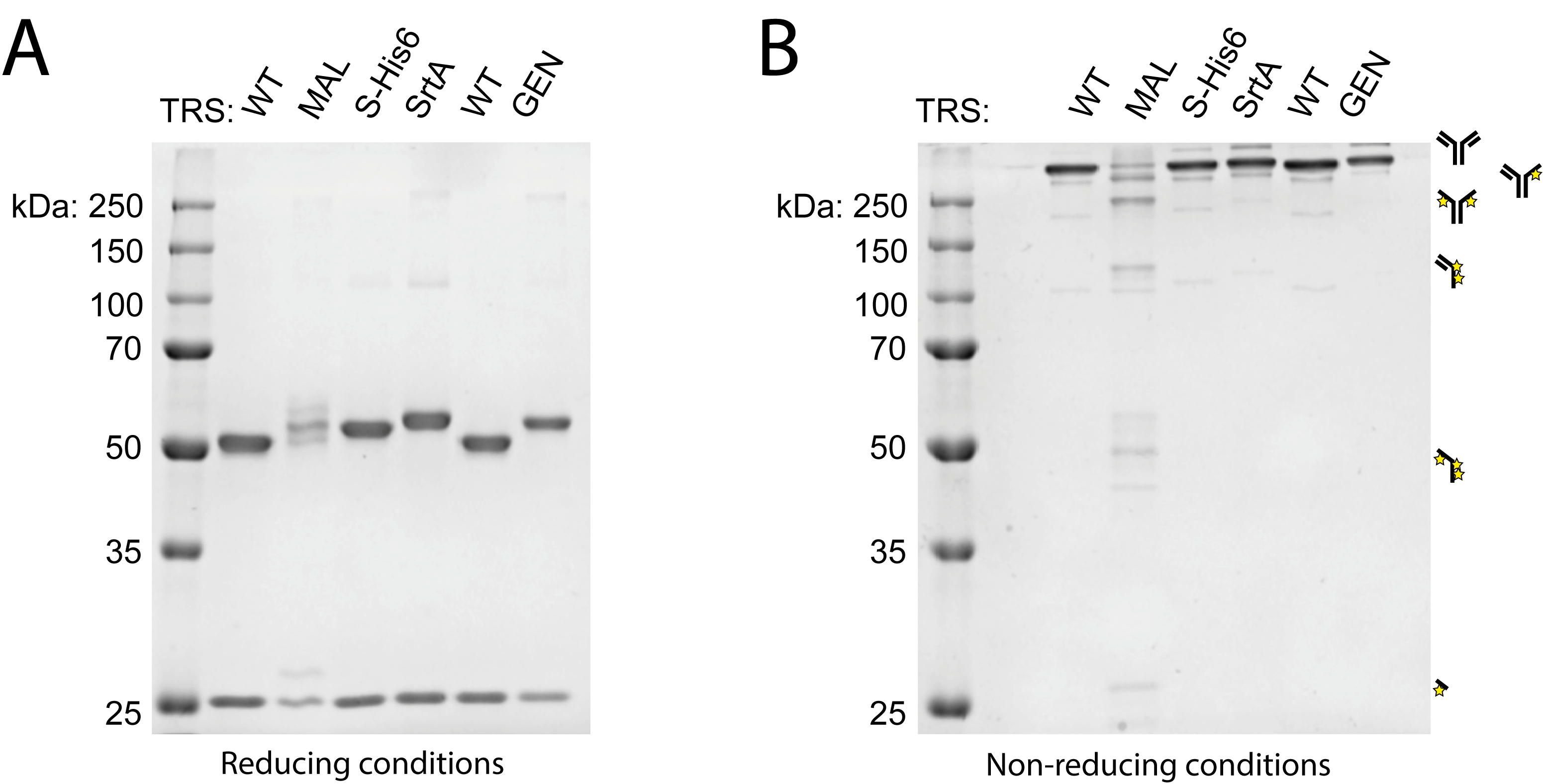

Supplement: Supplementary Figure 3 — An EBV epitope preceded by a protease cleavage sequence can be conjugated chemically, enzymatically, or genetically to TRS. (A) All three the different conjugation strategies analysed with SDS-PAGE visualized with an instant blue staining under reducing and (B) non-reducing conditions. With the conjugation possibilities for CTX-MAL depicted next to the gel. The conjugation possibilities were determined by looking at the most abundant bands. [file Image_3.tif]

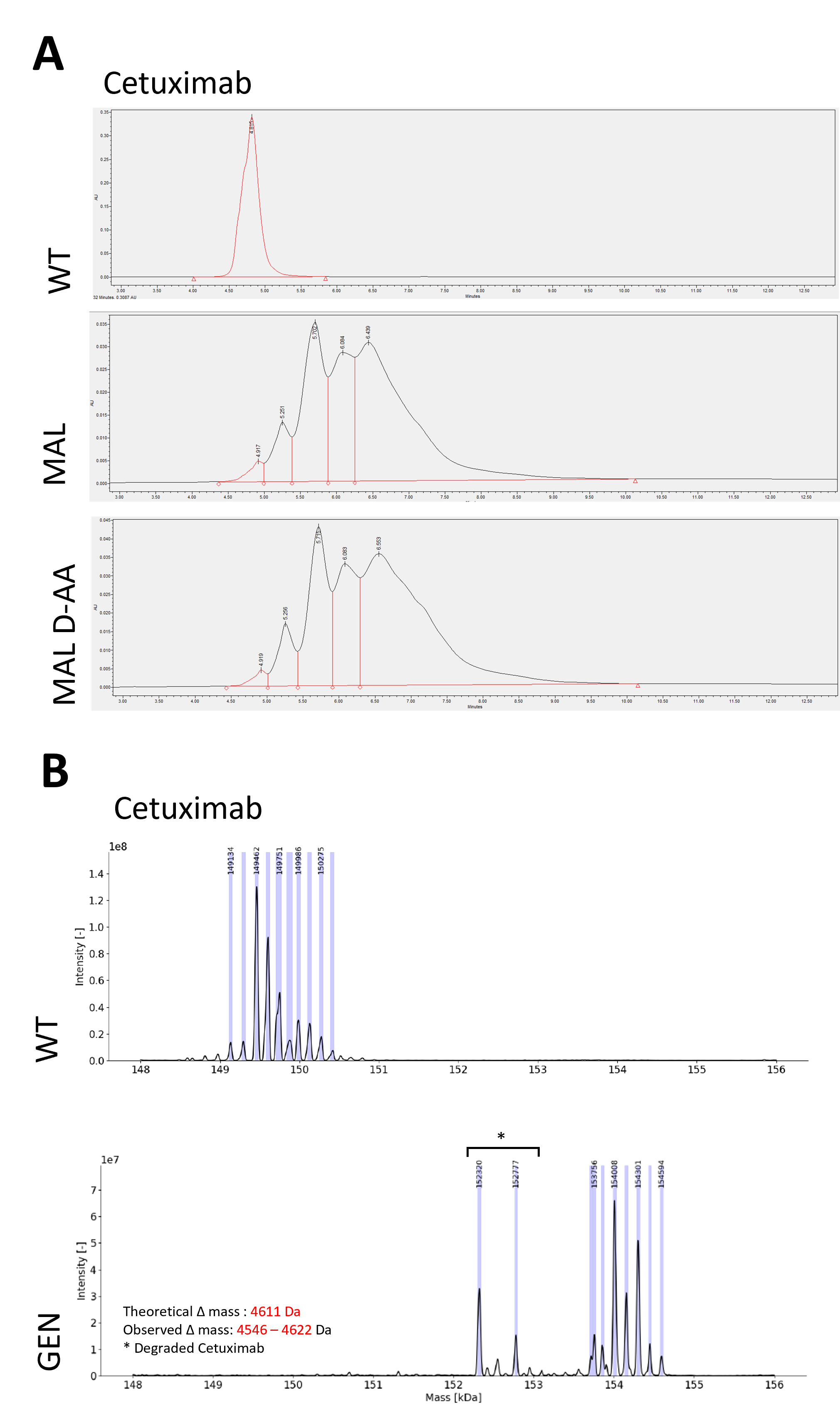

Supplement: Supplementary Figure 4 — Mass-spec and HIC analysis. (A) HIC analysis of the CTX-MAL conjugates and (B) the intact Mass-spec analysis of the CTX-GEN. [file Image_4.tif]

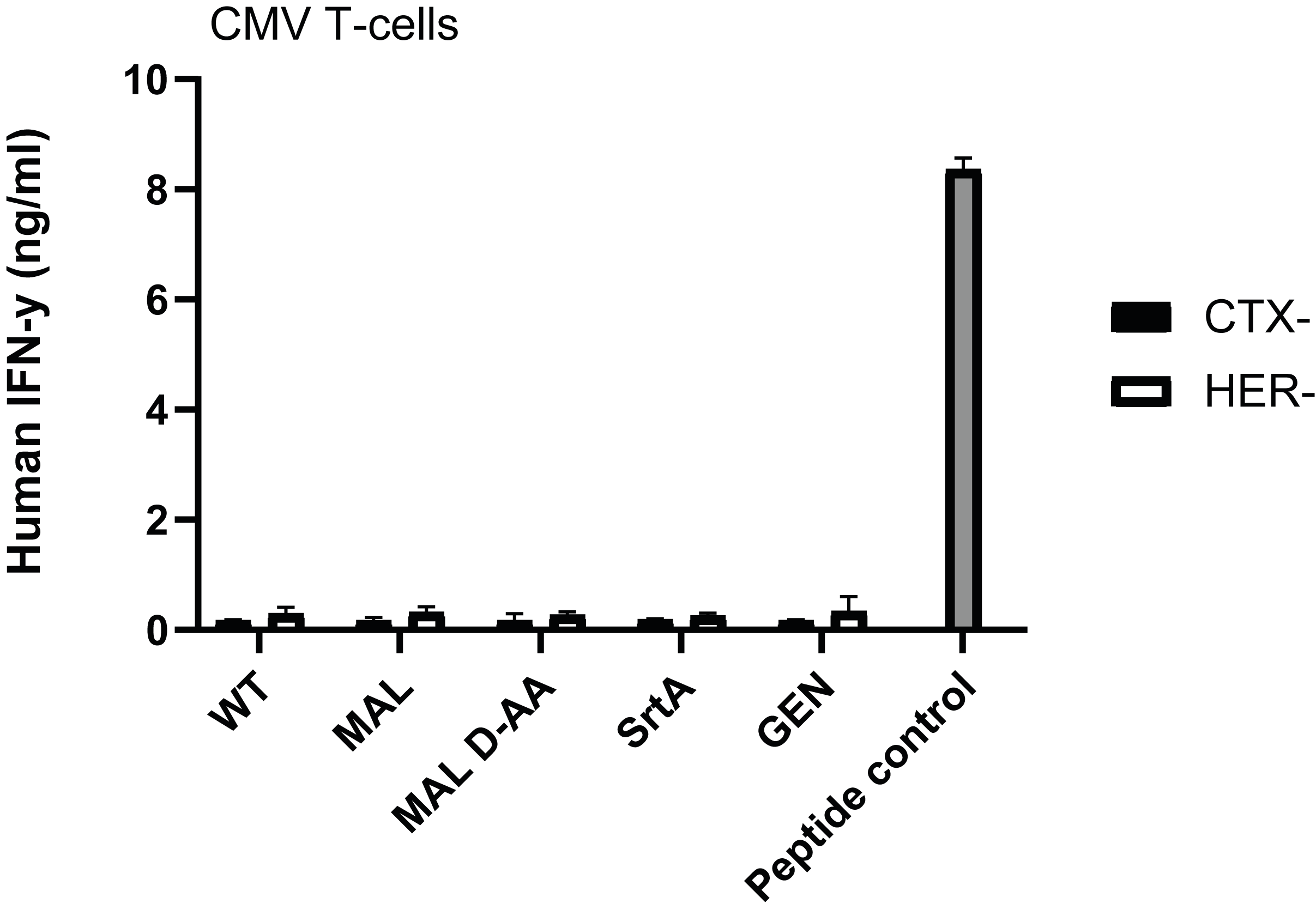

Supplement: Supplementary Figure 5 — The CTX- and TRS-AECs are not recognized by a non-specific CMV pp65/A2 specific T-cell line . HeLa-A2 ther2 cells were incubated with 16 nM of the different AECs, followed by a coculture with a CMV pp65/A2 specific T-cell line. T-cell activation was measured with an IFN-y ELISA. As a positive control, cells were incubated with the pp65/A2 peptide. [file Image_5.tif]

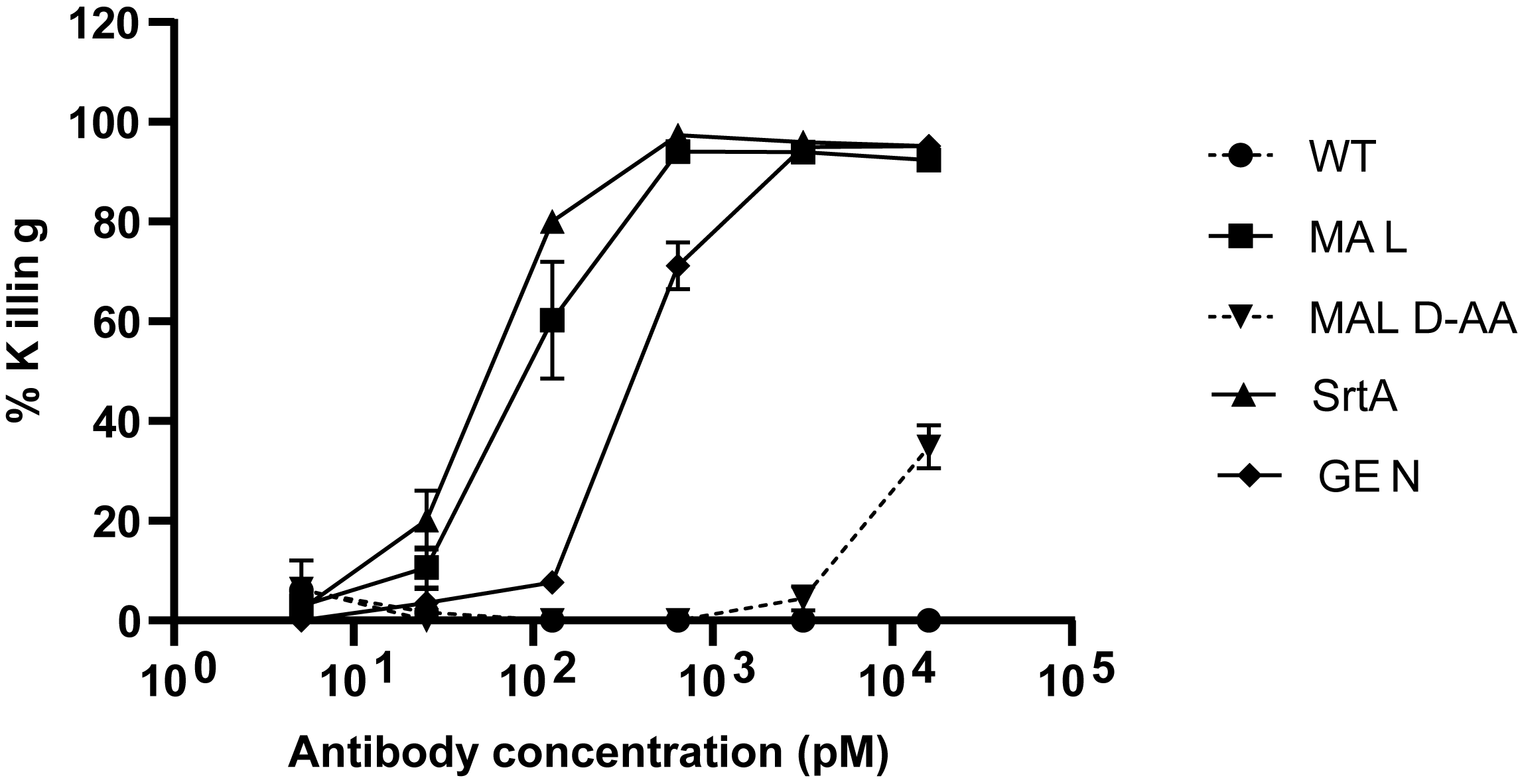

Supplement: Supplementary Figure 6 — Target cell killing is also observed for HeLa-A2 tHer2 incubated with the different TRS-AECs. To check for target cell killing, an AlamarBlue assay was performed. Plotted values are means of duplicates (SEM) and the shown graph is a representative figure of an n>3. [file Image_6.tif]

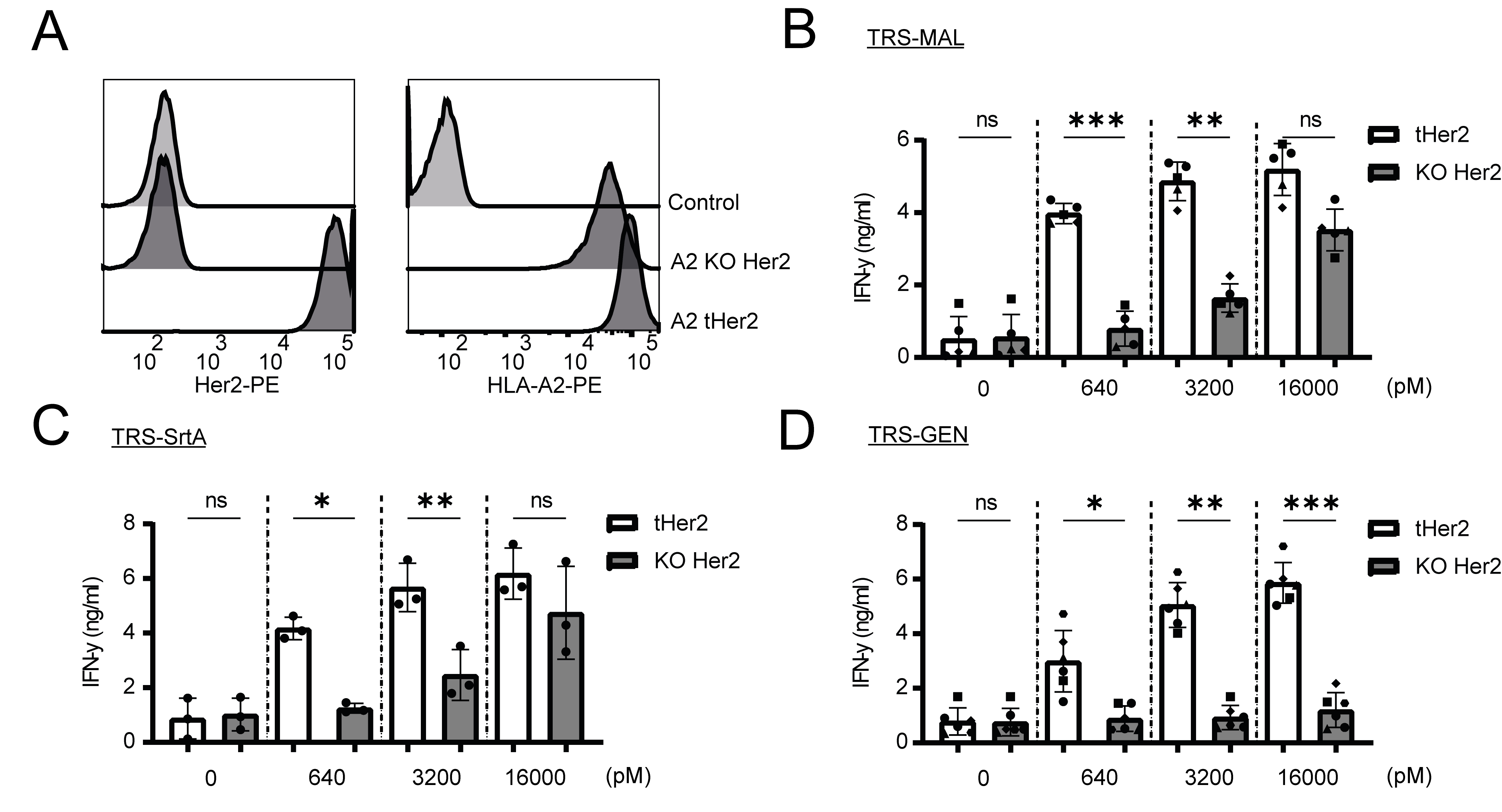

Supplement: Supplementary Figure 7 — Recognition and activation by the T-cells is target specific but show at high concentration differences between the conjugation strategies also for Trastuzumab. (A) KO cells were generated by using CRISPR/Cas9 and analysed with FACS for Her2 and HLA-A2 expression (B-D) All three TRS conjugates were titrated on the HeLa KO Her2 alongside the HeLa-A2 tHer2 and T-cell activation was measured with an IFN-y ELISA. Plotted values are the means of duplicates within one experiment of at least three independently performed experiments. For the statistical analysis a RM one-way ANOVA with Sidak multiple comparisons was performed. [file Image_7.tif]

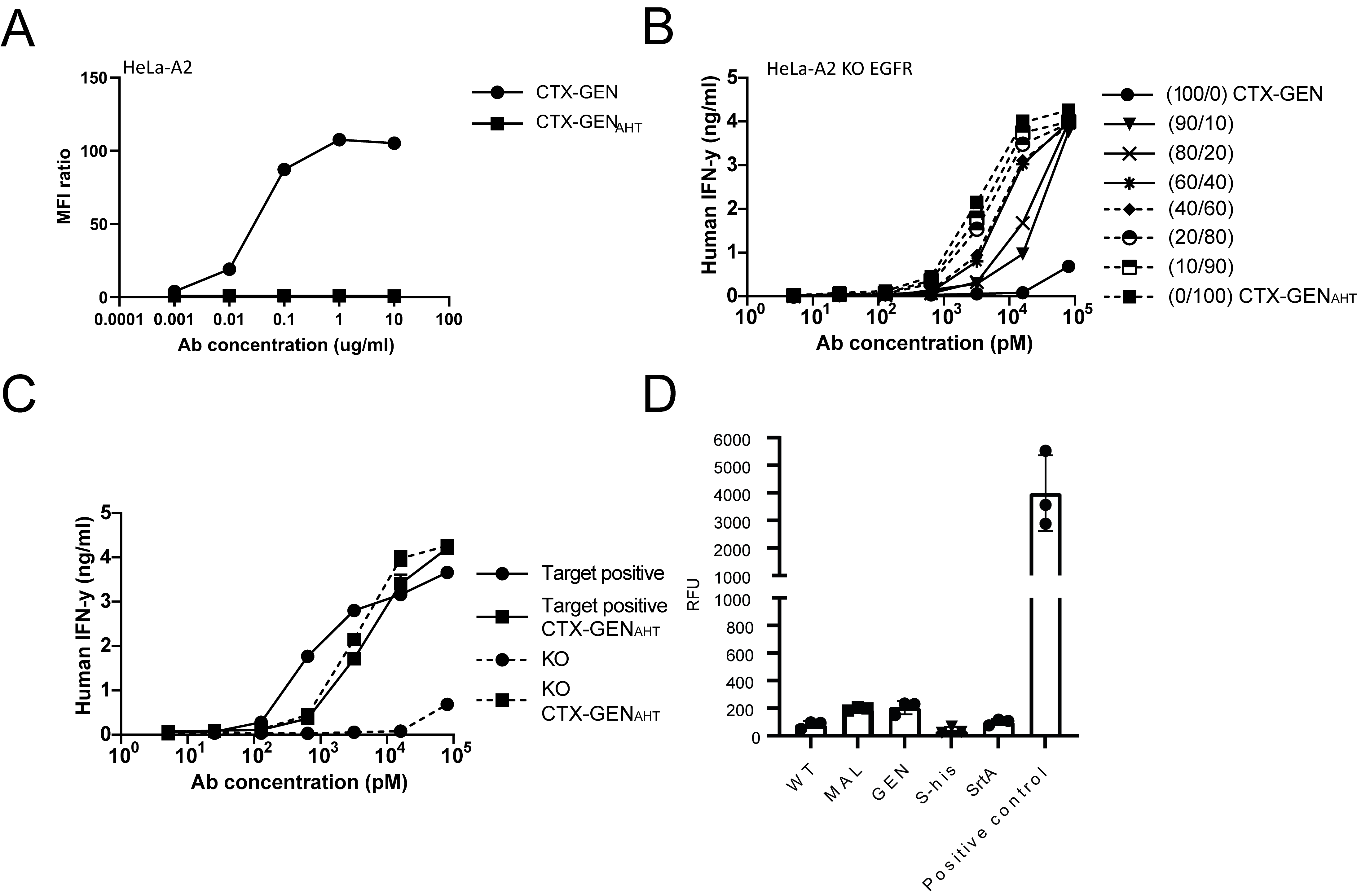

Supplement: Supplementary Figure 8 — Denatured AECs can result in T-cell activation independent of the antibody target. (A) CTX-GEN and CTX-GENAHT were titrated in an FACS experiment to check whether the treated antibodies were still able to bind (B) CTX-GEN and CTX-GENAHT were incubated on HeLa-A2 (target positive) and HeLa-A2 EGFR KO (target negative) cell lines. (C) CTX-GEN was mixed with CTX-GENAHT in different ratios (v/v) and incubated on the HeLa-A2 EGFR KO cell line. (D) Antibody aggregation was measured for the TRS conjugates with the PROTEOSTAT protein aggregation assay, in which the positive control is a Sortase A conjugate that is already known to aggregate. Plotted values are the means of duplicates within one experiment of at least three independently performed experiments. [file Image_8.tif]
