# Supplementary material for: Comparison of methods generating antibody-epitope conjugates for targeting cancer with virus-specific T cells
Source: Front Immunol. 2023 May 16;14:1183914. doi: 10.3389/fimmu.2023.1183914 (PMC10227578; doi:10.3389/fimmu.2023.1183914)
Supplement: Supplementary file 9 [file Table_1.pdf]

Supplementary Table 1: Overview of the amount of % conjugated antibody and the Epitope-to-antibody ratio

	CTX		TRS	
	Conjugated Ab (%)	EAR	Conjugated Ab (%)	EAR
**MAL (HIC)**	97.9	>2	95.4	>2
**SORT (SDS PAGE)**	-	2	-	2
**GEN (Mass-spec)**	-	2	-	2
